# Identification of an epidermal keratinocyte AMPA glutamate receptor involved in dermatopathies associated with sensory abnormalities

**DOI:** 10.1097/PR9.0000000000000573

**Published:** 2016-10-31

**Authors:** David Cabañero, Takeshi Irie, Marta Celorrio, Christopher Trousdale, David M. Owens, David Virley, Phillip J. Albrecht, Michael J. Caterina, Frank L. Rice, Jose A. Morón

**Affiliations:** a Department of Anesthesiology, Washington University Pain Center, Washington University School of Medicine, St Louis, MO, USA; b Department of Experimental and Health Sciences, University Pompeu Fabra, Barcelona, Spain; c Department of Anesthesiology and Critical Care, Memorial Sloan Kettering Cancer Center, New York, NY, USA; d Department of Dermatology, Columbia University Medical Center, New York, NY, USA; e Department of Pathology and Cell Biology, Columbia University Medical Center, New York, NY, USA; f Transpharmation Ltd, London Bioscience Innovation Centre, London, United Kingdom; g Integrated Tissue Dynamics, Rensselaer, NY, USA; h Department of Neurosurgery, Neurosurgery Pain Research Institute, Johns Hopkins School of Medicine, Baltimore, MD, USA; i Department of Neuroscience, Washington University School of Medicine, St Louis, MO, USA

**Keywords:** Keratinocytes, AMPAR, Glutamate, Pain

## Abstract

**Introduction:** Epidermal keratinocytes are increasingly recognized as active participants in the sensory transduction of itch and pain, processes known to involve primary afferent glutamatergic neurons. However, the role of keratinocyte glutamate signaling in sensory functioning is not fully understood. Here, we present the observation of α-amino-3-hydroxy-5-methyl-4-isoxazole-propionic acid–type glutamate receptors (AMPARs) in epidermal keratinocytes.

**Methods:** Immunohistochemical and in situ hybridization analyses were conducted to assess the expression of AMPAR subunits in epidermal keratinocytes in mouse and human skin samples, and in organotypic cultures of human keratinocytes. In addition, reverse transcription PCR further confirmed the expression of GluA4-containing AMPAR in epidermal keratinocytes.

**Results:** We found prominent immunolabeling for the GluA4 subunit of AMPAR in keratinocytes of glabrous and hairy skin of mouse epidermis, as well as in human epidermal keratinocytes. Reverse transcription PCR confirmed Gria4 transcript expression in epidermal mouse keratinocytes. In addition, expression of GRIA4 mRNA was confirmed in epidermal human keratinocytes by in situ hybridization. Immunohistochemical studies conducted in human skin biopsies from patients with atopic dermatitis and postherpetic neuralgia demonstrate that keratinocyte expression of GluA4 can be altered under pathological conditions. Moreover, a decrease of GluA4 expression was observed in organotypic cultures of human keratinocytes after direct application of algogenic agents.

**Conclusion:** We provide evidence that GluA4-containing AMPARs are expressed in epidermal keratinocytes, that human pruritic and painful dermatopathologies have alterations in the keratinocyte expression levels of GluA4-containing AMPAR, and that itch- and pain-producing substances can directly regulate their production in keratinocytes.

## Abstract 2

Supplemental Digital Content is Available in the Text.

This study presents the discovery of AMPA glutamate receptors in mouse and human epidermal keratinocytes and their potential role in the physiopathology of itch or pain.

## 1. Introduction

The epidermis, the outermost layer of the skin, constitutes a critical barrier between our internal and external environments, participates in thermoregulation and innate immunity, and is innervated by primary afferent neurons that transmit sensory information including touch, itch, and pain.^22,43^ Keratinocytes are the most abundant cell type in the epidermis, and exhibit direct responsiveness to a wide range of physical and chemical stimuli.^38^ They are also located in close physical proximity to the peripheral intraepidermal nerve fiber (IENF) termini of C- and Aδ-type fibers, and peptidergic termini can release a host of neurochemical mediators on stimulation.^1^ Accordingly, a growing body of data suggest the existence of reciprocal interactions between keratinocytes and epidermal sensory nerve termini.^33,36^ However, the ability of keratinocytes to respond to classical neurotransmitters and other intercellular signaling molecules is incompletely understood, as is the role of keratinocytes in pathological sensory states.

In the central nervous system, glutamate is the main excitatory neurotransmitter, and α-amino-3-hydroxy-5-methyl-4-isoxazole-propionic acid–type glutamate receptors (AMPARs) are the primary excitatory ion channels for fast neurotransmission across synapses. A functional AMPAR is a tetramer consisting of dimers assembled from 4 possible protein subunits: GluA1, GluA2, GluA3, and GluA4,^37,48^ which are encoded by the genes *Gria1*, *Gria2*, *Gria3*, and *Gria4* (*GRIA1-4* in humans). Although expression of AMPAR has been extensively studied in the central nervous system,^48^ they have also been observed in peripheral nerves, where they are up-regulated in painful conditions.^10,11^ These AMPARs have been described to be functional in in vivo pharmacological experiments, wherein activation and inhibition produce pronociceptive and antinociceptive effects, respectively.^19,53^

Prompted by our recent findings showing that spinal cord GluA4-containing AMPAR and C-fibers innervating the skin are involved in opioid-induced pain,^8,24^ we sought to explore in greater detail GluA4 AMPAR expression in the primary afferents innervating the glabrous skin of the mouse. Unexpectedly, we found prominent GluA4 immunolabeling (GluA4-IL) in epidermal mouse keratinocytes which was confirmed by detecting GluA4 mRNA by reverse transcription PCR (RT-PCR) of fluorescence-activated cell sorting (FACS)-isolated mouse keratinocytes. Immunohistochemical and in situ hybridization analyses also revealed GluA4 expression in keratinocytes in human skin. Moreover, we observed an increase in keratinocyte GluA4 expression in skin biopsies from patients afflicted with atopic dermatitis (AD), while a decrease in GluA4 was observed in postherpetic neuralgia (PHN).^25,30,31,35^ Furthermore, a decrease of GluA4 expression occurred in organotypic cultures of human keratinocytes treated with algogenic agents. Collectively, this study documents for the first time the expression and regulation of AMPAR in epidermal keratinocytes, and suggests a critical role for GluA4 AMPAR in 2 clinical conditions involving chronic itch and pain.

## 2. Methods

### 2.1. Animals

Eight to 9-week-old C57BL/6 male mice were used. Protocols were approved by the Institutional Animal Care and Use Committee at Columbia University in New York, and Washington University in St Louis and met the guidelines of the National Institutes of Health's Guide for the Care and Use of Laboratory animals (Department of Health, Education, and Welfare publication no. 85-23, revised 1985, USA).

### 2.2. Mouse tissue preparation for immunofluorescence

Glabrous skin from the hind paw and hairy skin from the back of the mouse were depilated with Surgi Cream-Extra Gentle for Face (Ardell, Los Angeles, CA) and removed by blunt-dissection. Skin pieces were spread on an index card with the epidermal surface up, fixed in 4% paraformaldehyde (PFA, Sigma-Aldrich, Saint Louis, MO) for 20 minutes, washed 3 times in phosphate-buffered saline (PBS) for 5 minutes/wash, then sunk in 30% sucrose in PBS for 24 hours. The skin was removed, embedded in Tissue-Tek optimum cutting temperature (OCT) compound (Sakura Finetek, Torrance, CA), and frozen. Samples were cut in a cross-sectional plane at 25 μm thicknesses using a Microm HM 525 Cryostat (Thermo Scientific, Waltham, MA) and were thaw mounted onto Fisherbrand Superfrost Plus microscope slides (Fisher Scientific, Pittsburgh, PA). Slides with sections were kept at −80°C until use.

### 2.3. Immunofluorescence microscopy

Samples were permeabilized and blocked using 5% normal goat serum/Triton X-100 (NGST) applied directly over the microscope slide for one hour. Five percent NGST was made in PBS containing 0.3% Triton X-100 and 5% normal goat serum or 5% normal donkey serum (Sigma-Aldrich). After permeabilization, samples were incubated with primary antibody overnight in 1% NGST, then washed 3 times in fresh PBS (5 min/wash). After 1 hour incubation with the secondary antibody in 1% NGST, the slides were washed again 5 times (10 min/wash), and extra-fine coverslips (Fisherfinest, Premium Cover Glass; Fisher Scientific Pittsburgh, PA) were mounted using Fluoromount G mounting media (SouthernBiotech, Birmingham, AL). Samples were visualized using an Eclipse Ti confocal microscope (Leica Microsystems Inc., Buffalo Grove, IL).

### 2.4. Human newborn foreskin samples

Samples were kindly provided by the Cell and Tissue Kinetics Core of the Skin Disease Research Center at Columbia University Medical Center (New York) and also were obtained from St Louis Children's Hospital at Washington University School of Medicine (St Louis). The foreskins were collected from routine circumcisions performed on neonates at the New York-Presbyterian/Weill Cornell Medical Center (New York, NY) and Children's Hospital in St Louis, MO. Samples were unfixed and cryoembedded in OCT compound.

### 2.5. Fluorescence-activated cell sorting

Primary adult epidermal keratinocytes were isolated from the dorsal skin of 8- to 9-week-old mice as previously described.^39^ Freshly isolated epidermal keratinocytes were suspended in 1% bovine serum albumin (BSA)-PBS and stained with phycoerythrin (PE)-conjugated CD49f-Integrin α6, fluorescein isothiocyanate (FITC)-conjugated Ly-6A/E (Sca-1), and allophycocyanin (APC) conjugated CD34 antibodies (see below for details regarding dilutions). After washing, cells were stained with 4′,6-Diamidino-2-phenylindole dihydrochloride (DAPI) and then subjected to FACS analysis using a FACS Aria sorter and FACS DiVa 4.1 software (BD Biosciences, San Jose, CA). In all cases, cells flew under 30 psi pressure through a 100 μm nozzle. After eliminating DAPI-positive dead cells, FITC, APC, and PE signals were collected through 530/30, 660/20, and 576/26 nm band-pass filters, respectively. Viable α6-positive cells (PE+) were identified and further gated based on their surface expression of CD34 (APC+) and the absence of Sca-1 (FITC−).

### 2.6. Antibodies

The following primary antibodies were used and combined according to species compatibility: rabbit anti-GluA4C, targeting the C-terminus of GluA4 (1:250, #AB1508; EMD Millipore, Billerica, MA), guinea pig anti–GluA4N, targeting the N-terminus of GluA4 (1:50 for immunohistochemistry #GluA4N-GP-Af640; Frontier Institute Co, Ltd, Hokkaido, Japan), Alexa647-conjugated rat anti–CD34 (1:50 for immunohistochemistry; 1:100 for FACS, #560233; BD Biosciences, San Diego, CA), PE-conjugated rat anti–CD49f-integrin α6 (1:100, #555736; BD Biosciences), FITC-conjugated rat anti–Ly-6A/E (Sca-1) (1:100, #11-5981-81; BD Bioscience), rabbit anti–Loricrin (1:1000, #PRB-145P; BioLegend, Dedham, MA), rabbit anti–thymic stromal lymphopoietin (TSLP) (1:250, #ab47943; Abcam Inc, Cambridge, MA). Alexa Fluor–conjugated secondary antibodies were all obtained from Invitrogen-Molecular Probes (Waltham, MA), and used at 1:1000 (#A11008 Alexa Fluor 488 goat anti–rabbit (H+L), #A21450 Alexa Fluor 647 goat anti–guinea pig, and #A11005 Alexa Fluor 594 goat anti–mouse (H+L)). In addition to validation by mRNA expression, IL experimental controls included omission of primary antibodies.

### 2.7. Mouse keratinocyte reverse transcription polymerase chain reaction

Total RNA was harvested from 2.5 to 9 × 10^6^ cells of interest by TRIzol (Life Technologies, Waltham, MA)/chloroform extraction with an RNeasy kit (Qiagen, Valencia, CA). RNA was quantitated using a Nanodrop (ND-1000 Thermo Scientific), and PCR reactions were prepared with 25 ng/12.5 μL OneStep (Qiagen #210210) RT-PCR reaction using oligos from IDT DNA with the following sequences (5′–>3′): *Gapdh*_ex2-F ATGGTGAAGGTCGGTGTGA, *Gapdh*_ex3-R TTTGATGTTAGTGGGGTCTCG, *CD34*_ex1-F GCTCTCTGCCTGATGAGTCTG, CD34_ex3-R CCTTAATGGCACTCGGAGC, *Gria4*_ex18-F GAGTGCCTTGAGCCTGAGC, and *Gria4*_ex19-R GGTAGGTCCGATGCAATGAC. All PCR oligos were designed to span introns to avoid amplification of contaminating genomic DNA. The RT-PCR strategy for *Gria4* followed this scheme: RT with 50°C 30 minutes and deactivation at 95°C 15 minutes, followed directly by touch-down PCR with initial cycle conditions of 94°C 30 seconds, 68°C 30 seconds, 72°C 20 seconds, with annealing temperatures decremented 1°C/cycle to a low of 60°C, followed by 30 more amplification cycles with 60°C annealing, and a 72°C 10 minutes final extension step. *Gapdh* and *CD34* amplifications were performed without touchdown, using the following scheme: RT with 50°C 30 minutes and deactivation at 95°C 15 minutes, followed directly by PCR with cycle conditions of 94°C 30 seconds, 54°C 30 seconds, 72°C 30 seconds, and a 72°C final extension step. PCR products were separated on a 1.5% agarose/TAE gel, and bands of interest were excised and purified with a Qiaquick gel extraction kit (Qiagen #28704). Recovered PCR products were quantitated by Nanodrop (Thermo Scientific, Wilmington, DE), and Sanger sequenced. Sequence AB1 files were visualized with 4Peaks software (Mek&Tosj.com, Amsterdam, the Netherlands).

### 2.8. Human skin biopsies

Potential alterations in GluA4, TSLP, and Loricrin expression were assessed by immunofluorescence in skin biopsies from human patients afflicted with chronic itch-associated AD and patients having chronic pain associated with PHN. Skin punch biopsies 5 mm in diameter taken from forearms of 3 patients diagnosed with AD in the biopsy locations were purchased from a commercial supplier (Astrand Ltd) (Table 1). Two biopsies were obtained from patient AD 1: one from a nondiseased (“normal”) skin site and one from a site on the forearm afflicted with AD. The other 2 patients (AD 2 and AD 3) were each the source of a single biopsy; one from an AD-afflicted site on the arm (AD 2) and one from an AD-afflicted site on the back (AD 3). These biopsies were flash frozen in isopentane chilled with liquid nitrogen, stored at −80°C, and shipped on dry ice by overnight courier for histological processing.

**Table 1:**
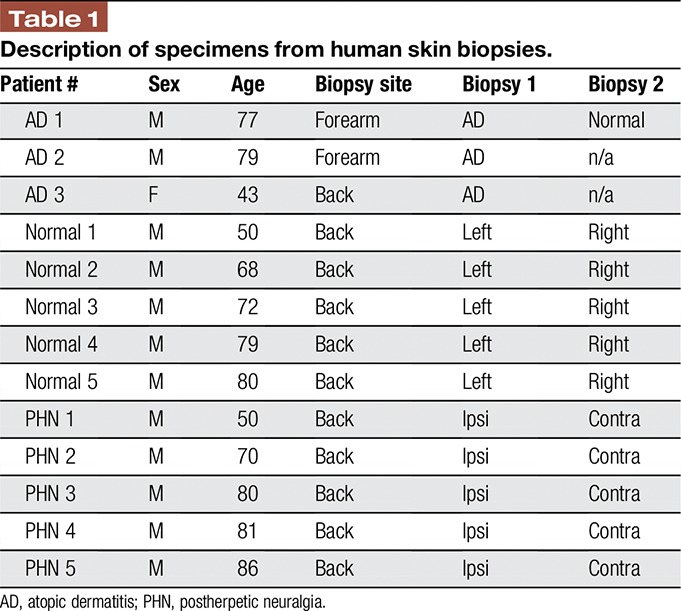
Description of specimens from human skin biopsies.

Following procedures approved by the Albany Medical College Institutional Review Board, four 3-mm punch biopsies of back skin were obtained from each of the 5 “normal” subjects (Nor 1–5) with no known neuropathic or dermatopathic conditions and each of the 5 patients (PHN 1–5) who resolved with PHN after being afflicted with acute herpes zoster in a unilateral thoracic dermatome (Table 1). Each PHN patient had a pain rating >3/10 on a visual analog scale. Two closely spaced biopsies were taken from each mirror-image location on the right and left dermatomes of the normal subjects, and 2 each from the PHN-afflicted dermatome (ipsilateral) and from the mirror image locations on the unafflicted dermatome (contralateral). One biopsy from each collection location of each person was flash frozen in isopentane chilled with liquid nitrogen and stored at −80°C until sectioning. The second biopsy from each location was fixed by immersion for 4 hours in 4% PFA in 0.1M PBS at 4°C, pH 7.4. After fixation, the PFA immersion-fixed biopsies were rinsed in copious cold PBS and stored under refrigeration for histological processing. Before freezing and cutting, the immersion-fixed biopsies were cryoprotected by infiltration with 30% sucrose (Sigma-Aldrich) overnight.

The normal vs diseased status of the biopsies was blinded before processing and remained so during subsequent analyses. All biopsies were sectioned at 14 μm thicknesses using cryostat at −20°C in a plane perpendicular to the epidermis. For the flash-frozen biopsies, sections were thaw-mounted onto slides (maintained in the cryostat during cutting) by briefly touching the underside of the slide with a finger tip. The slides were stored at −80°C until further processing. Various methods of fixing the flash-frozen sections on slide were assessed, with 10-minute immersion in −20°C acetone providing IL results most similar to that of PFA-fixed skin. Sections from PFA-fixed biopsies were thaw-mounted onto room temperature slides and air-dried overnight until further processing. Sections from all biopsies were mounted in serial order, rotated across 20 slides such that 10 slides had approximately 15 sections spaced at equal intervals through each biopsy and 10 others had 3 sections spaced at equal intervals through each biopsy. The slides were then used for different single and double IL combinations to develop a comprehensive immunocytochemical and morphological profile of each biopsy.

For IL, all slides were preincubated for 30 minutes at room temperature in PBS with 1% BSA. Incubations with primary antibodies diluted in PBS-BSA were conducted overnight under refrigeration, followed by 30-minute rinse in PBS and incubation with secondary antibodies diluted in PBS-BSA at room temperature for 2 hours. The slides were rinsed in PBS and coverslipped with 95% glycerin in PBS. Unused slides were hydrated with PBS and archived under glycerin-mounted coverslips at −20°C for future use with other biomarkers or repeat assessments as needed. For this study, sections were processed with double-label combinations of the guinea pig anti–GluA4N, rabbit anti–GluA4C, rabbit anti–Loricrin, and rabbit anti–TSLP as described. Secondary antibodies used were donkey anti–guinea pig IgG or donkey anti–rabbit IgG conjugated to Cy3 or Alexa 488. Sections were counterstained with DAPI to label cell nuclei. Several slides from these biopsies had previously been analyzed to assess characteristics of the innervation and other biomarkers among the epidermal keratinocytes which are not the subject of this study.

Epifluorescence images from the human biopsy samples and the organotypic epidermal cultures (see below) were captured using an Olympus BX51-WI microscope system equipped with a Hamamatsu ER high-speed camera, controlled via Neurolucida software (MBF Biosciences, Essex, VT) operating Win7 on a stand-alone PC. For all experiments, optimal camera capture settings were determined for each immunolabel before analysis, and the identical camera settings were used to capture all images of a given immunolabel across each experimental group.

### 2.9. Fluorescence in situ hybridization

Fluorescence in situ hybridization (FISH) analyses were conducted to confirm the results of the GluA4 IL analyses. The RNAscope Multiplex Fluorescent Assay (Advanced Cell Diagnostics [ACD], Hayward, CA) was performed using target probes to GRIA4 on alternating sections of the PFA-fixed human skin biopsies. Unfortunately, no alternating sections of the flash-frozen biopsies were available because of previous use in optimizing postsectioning fixation protocols for IL. The tissue sections were treated with pretreatment solutions of proprietary composition (ACD) and were used according to the manufacturer's instructions. The slides were washed with 0.1M PBS in a Tissue-Tek slide rack for 5 minutes to remove OCT. The tissues were pretreated sequentially with 1× Target Retrieval solution (or pretreatment solution 2) in a beaker at 99°C to 100°C for 5 minutes and with pretreatment solution 3 (protease digestion) 30 minutes at room temperature. The slides were then incubated with a custom-synthesized GRIA4 RNAscope probe for 2 hours at 40°C, followed by amplifying hybridization processes. Tissue sections were then incubated with Amp1 (preamplifier) for 30 minutes at 40°C, Amp2 (background reducer) for 15 minutes at 40°C, Amp3 (amplifier) for 30 minutes at 40°C, and Amp4 (label probe) for 15 minutes at 40°C in a HybEZ oven (ACD). After each hybridization step, samples were rinsed in 1× wash buffer twice for 2 minutes each at room temperature. The GRIA4 RNAscope human probe contains 1067 base pairs (NM_000829.3, ACD#300031). The slides were stained with DAPI for 30 seconds at room temperature and were then mounted with mounting medium for fluorescence (Vectashield; Vector laboratories, Burlingame, CA). Sections were imaged with a Leica scanning confocal microscope (Leica Microsystems Inc.) using an LD C-Apochromat 40× oil lens. Z stacks were performed on all sections using pinhole settings that resulted in 1.0-μm thick optical sections.

### 2.10. Quantification of GluA4 and *GRIA4* expression in epidermal keratinocytes

For the back skin biopsies of the 5 PHN patients and 5 normal subjects, the relative intensity of GluA4-IL in epidermal keratinocytes was assessed using Photoshop CS2 software (Adobe Systems, San Jose, CA) to calculate average pixel intensity at 8 equally spaced locations along the epidermis in each of the 3 sections spaced 1 quarter, half, and 3 quarters through the biopsy (ie, 24 samples or biopsy). A 2-tailed, paired *t* test was used to compare these 24 averages for the left and right biopsies from each normal subject and the ipsilateral and contralateral biopsies from each PHN patient.

### 2.11. Organotypic epidermal cultures

The StrataTest (Stratatech Inc, Madison, WI) 3D organotypic human skin cultures are composed of an epidermis and a dermis, manufactured using proprietary normal immortal keratinocytes (NIKS) and human dermal fibroblasts.^2^ The StrataTest 24 × 1 cm well-test plate provided fully stratified, multilayered human skin cultures in every well, and was shipped in gelatinized suspension media. On arrival, single 3D cultures were transferred to 6-well plates and placed into 1 mL of normal maintenance media for 24 hours to reconstitute viability, as per the manufacturer's instructions. Control cultures were then fed everyday for 3 consecutive days with normal maintenance media. Test cultures were fed on day 1 with normal maintenance media. On day 2, test cultures were fed with maintenance media that was supplemented with either 1 µM Capsaicin (Cap; Caymen Chemical, Ann Arbor, MI) in dimethyl sulfoxide or 1% (vol/vol) of complete Freund adjuvant (CFA; supplied as a 1 mg/mL suspension in nonmetabolizable oils [paraffin oil and mannide monooleate; Sigma-Aldrich]). The concentrations of the test substances carrier media (dimethyl sulfoxide or mineral oil) were considered negligible once the capsaicin or CFA were dissolved into the culture media. Test cultures had the test media replaced again on day 3 to create a 48-hour treatment period. On day 4, all cultures were rinsed 3× with warm PBS, fixed for 2 hour with ice-cold 4% PFA, rinsed and cryoprotected overnight in 30% sucrose at 4°C. Subsequently, cultures were frozen and cryostat sectioned perpendicular to the epidermal surface at 14 μm onto glass microscope slides for IL procedures as described above for skin biopsies.

## 3. Results

### 3.1. Mouse keratinocytes express GluA4 AMPAR subunits

**Figure 1.**
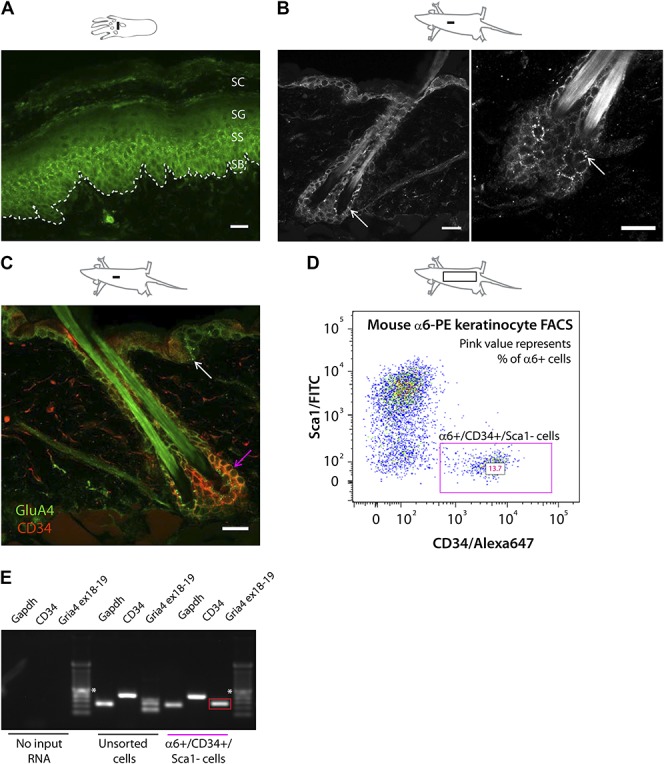
Mouse keratinocytes express the GluA4 AMPAR subunit. (A) In glabrous skin, confocal imaging revealed prominent GluA4-IL consistently among the suprabasal differentiating keratinocytes in SS of the epidermis. The labeling was more variable and generally less intense among the keratinocytes in SB and was consistently weak among the superficial differentiated keratinocytes of SG. Dotted line indicates dermal or epidermal basement membrane boundary. (B) In hairy skin, GluA4-IL (arrows) was observed in the bulge of the hair follicle (pink arrow), and intermittently in SB of the interfollicular epidermis (white arrow). GluA4-IL appeared more prominent in the inner layer of the bulge cells. (C) Colocalization of GluA4 (green) with CD34 (red), a membrane marker of hair follicle bulge stem cells. Scale bar = 20 μm. (D) GluA4-positive cells were collected as α6+/CD34+/Sca-1− cells using FACS. (E) *Gria4* RT-PCR product of expected size (288 bp) was observed in CD34+ cells, while unsorted keratinocytes produced 2 RT-PCR bands. *Gapdh* and *CD34* primers were included as positive controls. Asterisks represent 600 bp of a 100 bp ladder. Red square in image: *Gria4* ex18-19, 288 bp. FACS, fluorescence-activated cell sorting; FITC, fluorescein isothiocyanate; IL, immunolabeling; PE, phycoerythrin; RT-PCR, reverse transcription PCR; SB, stratum basalis; SC, stratum corneum; SG, stratum granulosum; SS, stratum spinosum.

AMPAR subunit IL was first investigated in the glabrous skin epidermis of mouse hind paws. We found prominent GluA4-IL in suprabasal keratinocytes (GluA4C, see Methods) at the level of the stratum spinosum (SS, Fig. 1A), where the IL puncta decorated cell boundaries of the keratinocytes. To examine whether other AMPAR subunits were also expressed, we conducted additional IL; however, antibodies to the GluA1, A2, or A3 AMPAR subunits failed to detect a reliable signal. Additional labeling evaluating the expression of GluA4C in hairy skin from the dorsum of the mouse revealed GluA4-IL in the bulge of the hair follicle (Fig. 1B, arrow), and intermittently in the interfollicular epidermis (Fig. 1C, white arrow). Hair bulge keratinocyte stem cells express the transmembrane protein CD34,^5^ and colabeling of GluA4 and CD34 was confirmed (Fig. 1C), although GluA4-IL was more apparent in the inner layer of the bulge cells. Negative controls in the absence of primary antibody in mouse glabrous and hairy skin were also run to test the specificity of the GluA4 labeling (Supplementary Figure 1A, available online at http://links.lww.com/PR9/A1).

To corroborate GluA4 expression in CD34(+) keratinocytes, *Gria4* RT-PCR was performed on FACS-purified CD34(+) keratinocytes. Bulge keratinocytes were gated based on their expression of α6 integrin and CD34, and the relative absence of Sca-1 (Fig. 1D), as previously reported.^29^ FACS gating of α6(+)/CD34(+)/Sca-1(−) cells revealed that these cells comprised 13% of the live keratinocyte population (Fig. 1D). Gria4 RT-PCR of α6(+)/CD34(+)/Sca-1(−) keratinocytes was conducted using primers spanning exon 18 to exon 19 of *Gria4* (*Gria4* ex18-19). *CD34* and *Gapdh* were amplified as positive controls (Fig. 1E). *Gria4* ex18-19 primers yielded products of the expected size (Fig. 1E, red box, *Gria4* ex18-19: 288bp), and the identity of this product as amplified *Gria4* ex18-19 was sequence-verified (Supplementary Figure 2, available online at http://links.lww.com/PR9/A1). The unsorted keratinocyte RNA produced 2 Gria4 RT-PCR bands (Fig. 1E). These results confirmed the expression of Gria4/GluA4 mRNA in mouse CD34(+) keratinocytes.

### 3.2. Newborn and adult human keratinocytes express GluA4 AMPAR subunits

**Figure 2.**
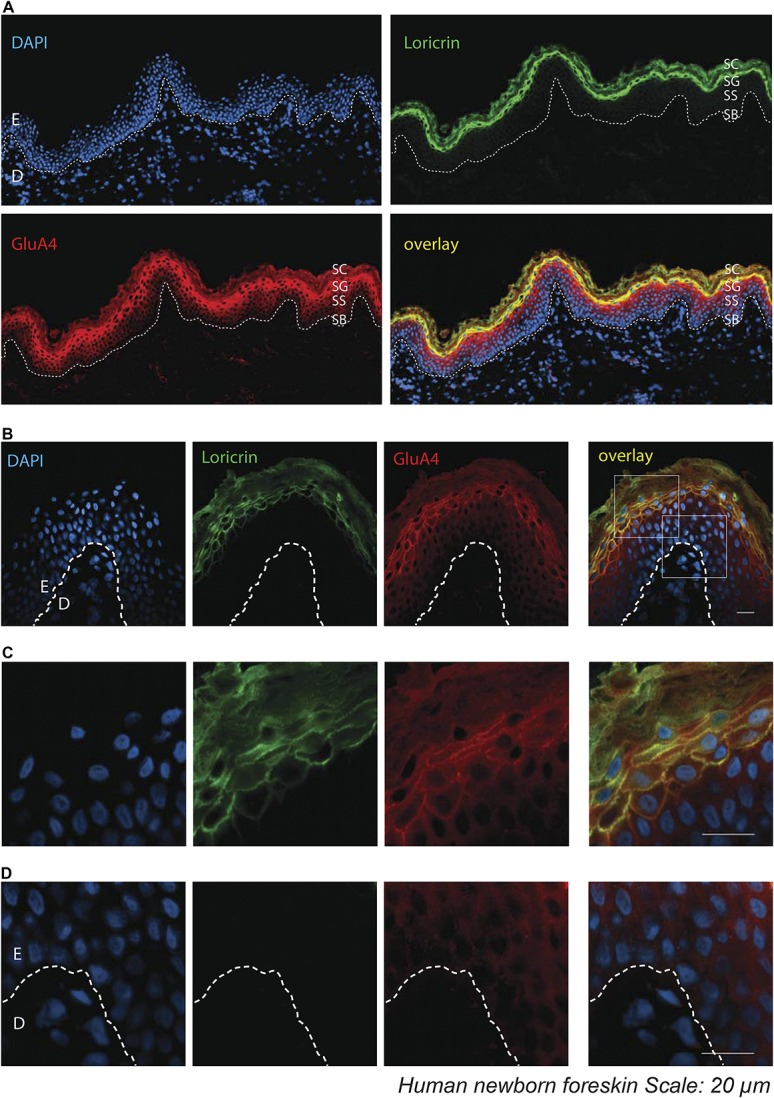
GluA4-IL is observed in epidermal keratinocytes from human newborn foreskin. GluA4-IL was present on keratinocytes in all the strata of the epidermis (A–D), especially in an apparent membranous localization among the suprabasal keratinocytes (C) and weakly and more intracellularly in SB (D). GluA4-IL was particularly concentrated on the keratinocytes of SG that colabel for Loricrin. Some residual GluA4-IL and Loricrin-IL are present on the dead keratinocytes in SC. DAPI was used to label the nuclei (blue), and the dotted lines indicate the epidermal (E)–dermal (D) boundary (insets from panel B overlay). Scale = 20 μm. IL, immunolabeling; SB, stratum basalis; SC, stratum corneum; SG, stratum granulosum; SS, stratum spinosum.

**Figure 3.**
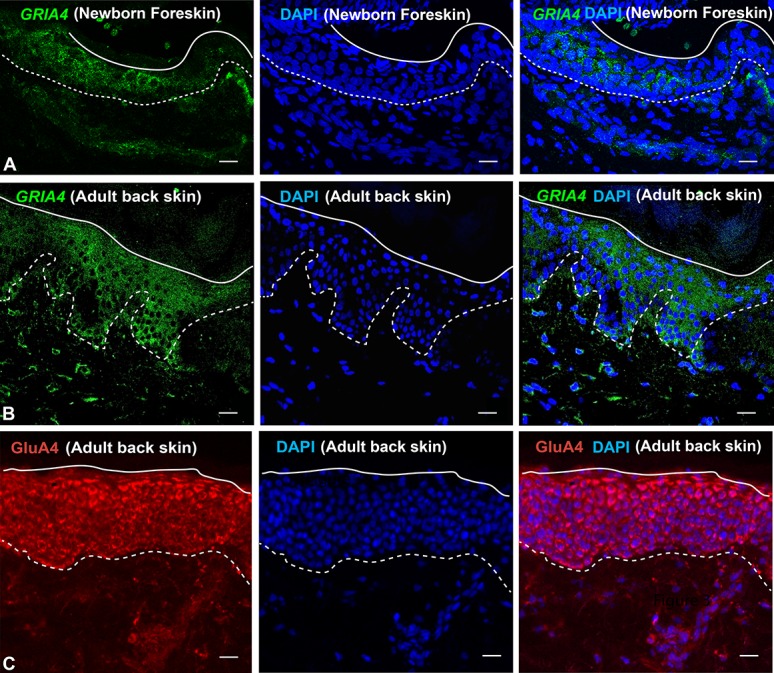
Expression of GluA4 protein and *GRIA4* transcript in human skin. Fluorescence in situ hybridization demonstrates GRIA4 mRNA expression among the epidermal keratinocytes of among keratinocytes of neonatal foreskin (A), and adult back skin biopsies (B). These results mirror the GluA4 protein expression in human back skin biopsies (C) and neonatal foreskin (Fig. 2). Representative ×40 confocal images for *in situ* hybridization detection of GRIA4 mRNA (green) are shown separate and merged with DAPI-labeled nuclei (blue). GRIA4 mRNA was observed in all the strata of the epidermis, but appeared particularly strong among SS or SG. Scale bar = 20 μm. SG, stratum granulosum; SS, stratum spinosum. Solid lines represent SC, dotted lines represent SB.

To evaluate potential conservation of the epidermal keratinocyte expression of GluA4, GluA4-IL was first investigated in sections of human newborn foreskin. GluA4-IL (GluA4N, see Methods) was observed through all the layers of the epidermis (Fig. 2A–D), prominently in the suprabasal layers (Fig. 2C) and weakly in the stratum basalis (SB, Fig. 2D). GluA4-IL was particularly concentrated among the keratinocytes of stratum granulosum (SG) which were colabeled with Loricrin, a protein whose expression is normally restricted to these terminally differentiated keratinocytes^9^ (Fig. 2A–C). Both GluA4-IL and Loricrin-IL appeared to be membrane-localized (Fig. 2C). Expression of GRIA4 transcript in human newborn foreskin was also investigated by FISH. Figure 3A and B demonstrates that the GRIA4 RNAscope human probe targeting base pairs 606–1673 showed robust signal among presumptive midlevel SS keratinocytes of the epidermis. The GRIA4 transcript expression among neonatal keratinocytes matches the expression observed for GluA4-IL, as seen in Figure 2A–D.

**Figure 4.**
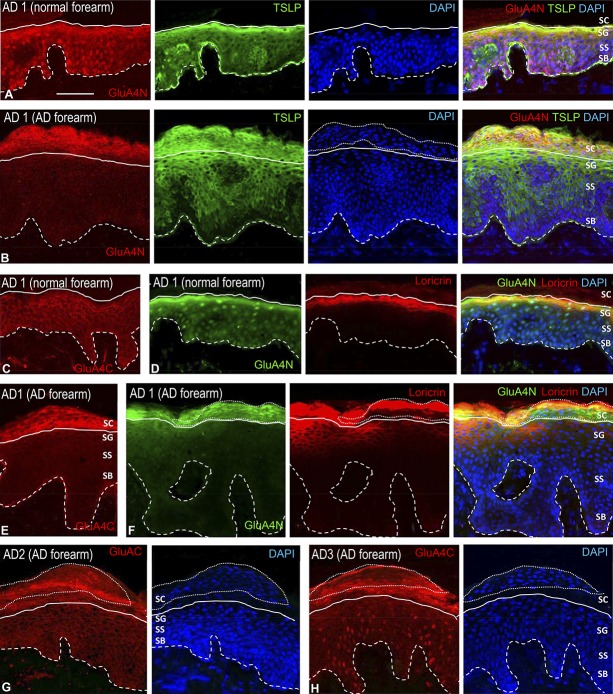
GluA4 expression is increased in skin biopsies from AD patients. Immunofluorescence labeling for GluA4, TSLP, and Loricrin among skin biopsies from a normal skin site and AD site from patient AD 1, and AD sites from patients AD 2 and AD 3. The sections are counterstained with DAPI to reveal cell nuclei. The dashed line indicates the location of the SB basement membrane dermal or epidermal junction; the solid line indicates the superficial border of SG which is normally the most superficial layer of vital nucleated keratinocytes; and the dotted line indicates zones of live keratinocytes that appear atopically located in the SC which is normally only composed of enucleated dead keratinocytes. Triple labeling for GluA4N (red), TSLP (green), and DAPI (blue) is shown in normal and AD skin. (A) In normal unaffected skin from patient AD 1, GluA4N-IL appears concentrated on the membrane and to a lesser degree in the cytoplasm of the most superficial keratinocytes of SG. Perinuclear GluA4N-IL is observed among deeper layer keratinocytes. TSLP-IL parallels the distribution of GluA4N-IL but is concentrated in the cytoplasm of the most superficial keratinocytes of SG with lower intensity expression in the cytoplasm of deeper keratinocytes. GluA4N-IL and TSLP-IL are faint or absent in SC. (B) In the AD biopsy from patient AD 1, GluA4N-IL is barely detectable among the live keratinocytes, but is intensely expressed in SC among the atopic live keratinocytes. By contrast, TSLP-IL is intensely expressed in the cytoplasm of keratinocytes throughout SG and most of SS, as well as in SC parakeratosis. (C) GluA4C-IL in unaffected control skin from AD 1 demonstrates expression among all the vital epidermal strata with higher expression among the SG keratinocytes, without evidence of perinuclear labeling. In the AD biopsy from patient AD 1, GluA4C-IL (E) is virtually identical to that of GluA4N-IL (B), particularly faint among the vital keratinocytes, but intense among the atopic parakeratosis. Triple labeling for GluA4N (green), Loricrin (red), DAPI (blue) in normal (D) and AD skin (F) revealed that in normal unaffected skin, Loricrin-IL is restricted to the most superficial keratinocytes of SG where there is the most intense expression of GluA4N-IL. In the AD biopsy, Loricrin-IL is expanded in SG and is also intensely expressed in SC. GluA4C-IL in the AD biopsies from patients AD 2 and AD 3 reveals that GluA4C-IL in patient AD 2 (G) has a membrane distribution among keratinocytes in most layers, much like that observed with the GluA4C-IL in the normal biopsy (C) but is also intensely expressed in SC. (H) The GluA4C-IL in patient AD 3 is mostly perinuclear among nucleated keratinocytes in SB, SS, and SG, and is also intensely expressed in SC. Scale bar = 100 μm. AD, atopic dermatitis; IL, immunolabeling; SB, stratum basalis; SC, stratum corneum; SG, stratum granulosum; SS, stratum spinosum; TSLP, thymic stromal lymphopoietin.

**Figure 5.**
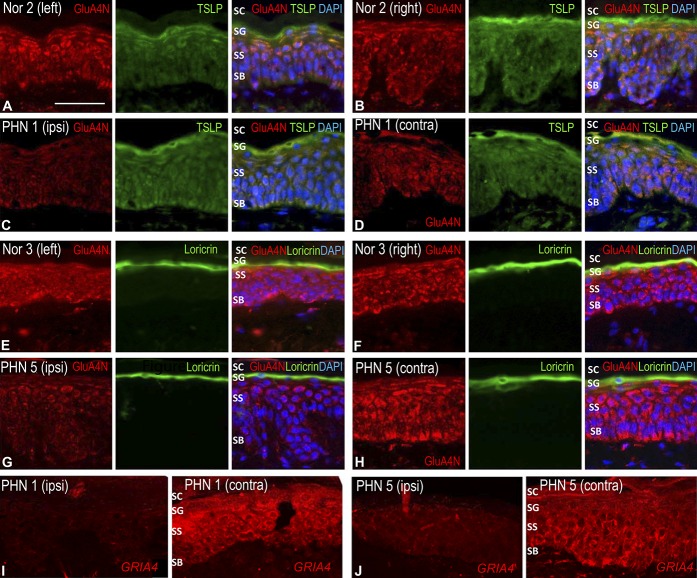
GluA4 expression is decreased in skin biopsies from PHN patients. (A) Immunofluorescence labeling for GluA4N, TSLP, and Loricrin as seen in 14-μm thick sections of PFA immersion-fixed back skin biopsies from normal human subjects and PHN patients. GluA4N is expressed on keratinocytes across SB, SS, and SG, but not in SC. In normal subjects, the intensity of GluA4N-IL is comparable in mirror-image biopsies from the left and right side (left panels in A, B and E, F). By contrast, GluA4N-IL intensity was consistently lower in the biopsies from PHN skin sites (ipsi) than in mirror-image contralateral unafflicted skin (contra, left panels C, D and G, H). (A–D) Triple labeling for GluA4N (red), TSLP (green), and DAPI (blue) in normal (A, B) and PHN skin (C, D). The TSLP-IL is expressed on keratinocytes throughout SB, SS, and appearing slightly more intense among the superficial keratinocytes in SG. The TSLP-IL appears to be slightly higher and more uniformly expressed among the keratinocytes in the PHN skin. (E–G) Triple labeling for GluA4N (red), Loricrin (green), and DAPI (blue) in normal (A, B) and PHN skin (C, D). Loricrin-IL is restricted to the most superficial keratinocytes of SG in normal skin and in both the PHN and mirror-image skin. (I, J) *GRIA4* mRNA expression in the ipsilateral PHN-afflicted and contralateral unafflicted dermatomes of patients PHN 1 and PHN 5. Scale bar = 100 μm. IL, immunolabeling; PFA, paraformaldehyde; PHN, postherpetic neuralgia; SB, stratum basalis; SC, stratum corneum; SG, stratum granulosum; SS, stratum spinosum; TSLP, thymic stromal lymphopoietin.

We then examined GluA4 expression in sections of punch biopsies of back skin from healthy adult volunteers (Fig. 3C), normal forearm skin from a patient with AD in other forearm locations, and back skin of patients with PHN taken contralateral to the dermatome injured by acute herpes zoster (Figs. 4 and 5). GluA4-IL was evaluated using 2 different GluA4 antibodies, GluA4N and GluA4C (see Methods). In the epidermis of normal adult back skin, GluA4-IL was observed among all epidermal vital keratinocyte layers, with most robust expression typically observed in outer SS and SG (Fig. 3C; Supplementary Figure 3, available online at http://links.lww.com/PR9/A1). As well, we also assessed the specificity of GluA4-IL in human biopsies from control volunteers (see Supplementary Figure 1B, available online at http://links.lww.com/PR9/A1). Furthermore, in situ hybridization on alternating sections from the same biopsies used to perform the IL confirmed expression of GRIA4 transcript in epidermal keratinocytes (Fig. 3B). In normal forearm epidermis, GluA4-IL increased from SB to SG (Fig. 4A, C). GluA4C-IL was mostly observed at or near the keratinocyte membrane (Fig. 4C), whereas GluA4N-IL appeared throughout the cytoplasm (Figs. 4D, 5A, B, E, F, H; Supplementary Figure 3, available online at http://links.lww.com/PR9/A1). In some specimens, both GluA4 antibodies produced labeling closely affiliated with the nuclei of keratinocytes in SS and SB (Fig. 5D, H). Together, these findings support a broad pattern of GluA4 protein expression in the adult epidermis.

In normal skin, Loricrin-IL was restricted to the most superficial keratinocytes of SG, overlapping with the outermost distribution of GluA4 labeling (Figs. 4D, 5E, F, H). GluA4-IL and Loricrin-IL were extremely faint among the cornified, enucleated keratinocytes of normal stratum corneum (SC) (Figs. 4D, 5E, F, H). Loricrin-IL was typically detected at or near the membranes of keratinocytes of healthy forearm glabrous skin (Fig. 4D), but exhibited cytoplasmic distribution in keratinocytes from skin of the back (Fig. 5F, H). In parallel with GluA4 and Loricrin, we also examined expression of TSLP, a cytokine implicated in AD.^15,50^ In both forearm and back skin epidermis of healthy skin, TSLP-IL expression coincided with GluA4-IL, with a similar expression gradient increasing from deep to superficial, with peak intensity in SG (Figs. 4A, 5A, B, D).

### 3.3. GluA4 expression in epidermal keratinocytes is altered in human dermatopathies associated with sensory abnormalities

Given our previous work on the altered expression of neurosignaling molecules in neuropathic pain conditions in humans,^25,52^ and our new evidence of GluA4 expression in human keratinocytes, we investigated GluA4-IL in the epidermis of patients with AD and PHN. Atopic dermatitis is the most common chronic inflammatory skin disease and manifests as overt rashes and clinically problematic itch that is not completely understood.^31,35^ Postherpetic neuralgia is a neuropathic pain condition occurring as a result of reactivation of varicella zoster virus, with initial nerve damage and rash, often followed by skin healing but recurrent severe allodynia and hyperalgesia at the rash site. In PHN, pathologies of epidermal nerve endings and keratinocytes remain long after remission of the initial rash associated with the primary zoster reactivation.^25,30^ Atopic dermatitis skin was compared with healthy skin within the same patient, and affected PHN skin was compared with unaffected contralateral skin from the same patients as well as with the skin from healthy patients.

Consistent with a previously known characteristic of AD,^45^ DAPI staining of sections from the biopsies from the afflicted sites in all 3 AD patients revealed plaques of nucleated keratinocytes in SC, referred to as parakeratosis^6^ (dotted outlines, Fig. 4B, F–H). Importantly, GluA4, Loricrin, and TSLP showed a distinct distribution pattern in all 3 biopsies from the AD patients. Intense GluA4, TSLP, and Loricrin-IL was present in SC of AD patients, particularly in the plaques where it far exceeded saturation at camera settings optimized for capture of GluA4 in biopsies of normal skin (Fig. 4B, F). GluA4-IL varied among the vital keratinocyte strata (SB, SS, and SG) of the biopsies from the 3 patients. Patient AD 1 had weak GluA4-IL across all the strata (Fig. 4E), while patient AD 2 had intense GluA4-IL among all the strata (Fig. 4G), whereas patient AD 3 had weaker membranous and stronger nuclear-like GluA4-IL scattered across all the strata (Fig. 4H). Compared with biopsies of normal skin locations, TSLP-IL in all 3 AD specimens was particularly robust within and near the membrane, as well as in the cytoplasm of keratinocytes in SS and SG (Fig. 4A, B for patient AD 1, not shown for patients AD 2 and AD 3). TSLP-IL remained faint in SB. Finally, Loricrin-IL was intense among SG keratinocytes, but was also expressed among SS keratinocytes, which is not observed in the control skin (Fig. 4D, F for patient AD 1, not shown for patients AD 2 and AD 3).

**Figure 6.**
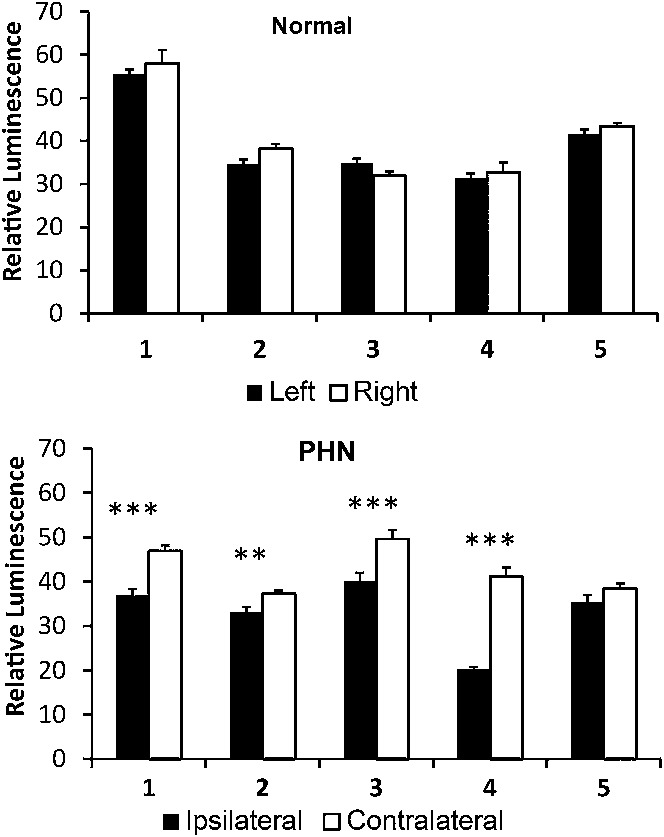
Quantification of the reduction in GluA4 levels in biopsies from postherpetic neuralgia (PHN) patients. Relative GluA4 immunolabel luminescence among epidermal keratinocytes of back skin biopsies taken from mirror-image locations in the left and right dermatomes of 5 normal subjects and PHN-afflicted (ipsilateral) dermatomes and unafflicted contralateral dermatomes of 5 PHN patients. **P < 0.01, ***P < 0.001.

GluA4-IL was also altered in skin of patients with PHN. We compared the relative intensity and distribution of GluA4-IL from dermatome-matched ipsilateral and contralateral biopsies from PHN patients and found that GluA4-IL from the afflicted sites was significantly diminished compared with dermatome-matched contralateral biopsy specimens in 4 of the 5 PHN patients (Figs. 5C, D, G, H, 6; Supplementary Figure 3, available online at http://links.lww.com/PR9/A1). These findings were confirmed using in situ hybridization, which also showed significantly lower GRIA4 transcript expression in the ipsilateral PHN compared with contralateral unafflicted epidermal keratinocytes within subjects (*P* > 0.005, paired *t* test) (Fig. 5I, J). The relative intensity and distribution of GluA4-IL were similar between the left and right dermatome-matched biopsies in all the 5 healthy subjects (Figs. 5A, B, E, F, 6; Supplementary Figure 3, available online at http://links.lww.com/PR9/A1). The GluA4-IL from biopsies of PHN patients taken from the contralateral sites demonstrated labeling comparable with that of the normal specimens (Figs. 5D, H, 6, Supplementary Figure 3, available online at http://links.lww.com/PR9/A1). TSLP-IL and Loricrin-IL were similar among the PHN patients and control subjects, including those from PHN-afflicted dermatomes (Fig. 5A–H).

### 3.4. GluA4 expression in organotypic keratinocyte cultures is modulated by algogenic substances

Glutamate signaling in keratinocytes is known to promote barrier functions, particularly mediated through NMDA receptors; however, a role for AMPAR in keratinocyte signaling has not been strongly established.^14,15^ AMPARs have been implicated in nociceptor hypersensitivity associated with chronic inflammatory pain mechanisms, and the use of peripheral AMPAR antagonists for inflammatory pain has been suggested previously.^16^ Keratinocyte signaling contributes to normal functioning and pathogenic pain mechanisms associated with inflammatory skin conditions; therefore, we adapted a standard model of inflammatory pain production from an in vivo setting CFA, as well as a known activator of keratinocytes (capsaicin) as test agents in human organotypic keratinocyte cultures to determine if these algogenic substances also affected the keratinocyte expression of GluA4.

**Figure 7.**
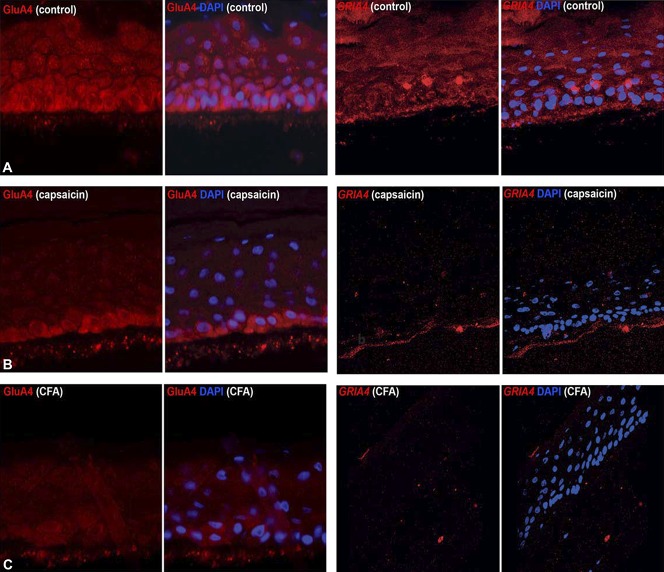
Organotypic cultures. GluA4-IL and GRIA4 mRNA expression with a DAPI counterstain in alternating sections from organotypic epidermal cultures exposed to (A) normal medium; (B) medium containing 1 micromolar capsaicin; and (C) medium containing 1% CFA. Both GluA4-IL and GRIA4 mRNA are severely depleted in the treated cultures. CFA, complete Freund adjuvant.

To best characterize the regulation of keratinocyte GluA4 expression, full-thickness epidermal cultures are essential, as monolayer keratinocytes do not fully differentiate into the signaling patterns observed in vivo. Therefore, we made use of organotypic cultures of fully differentiated and stratified epidermis generated from a stable immortalized line of human keratinocytes (Stratatech Inc, Madison, WI) and examined GluA4 expression by immunofluorescence and in situ hybridization after stimulation. As shown in Figure 7A, both GluA4 protein and GRIA4 mRNA were detected throughout the live layers of untreated keratinocytes. Capsaicin, a pungent chemical that binds the polymodal TRPV1 ion channel, evokes pain behaviors and can produce itch, has been reported to stimulate mediator release from isolated human keratinocytes.^34^ Treatment of the organotypic stratified keratinocyte cultures with capsaicin nearly eliminated GluA4 protein and *GRIA4* transcript detection among the suprabasal keratinocytes (Fig. 7B). Similarly, treatment of the cultures with CFA, a mycobacterial extract that induces pain behaviors and alters keratinocyte neurochemistry in vivo^25^ likely through toll-like receptor activation, also reduced the detection of both the GluA4 protein and *GRIA4* transcript in the cultures (Fig. 7C).

## 4. Discussion

Much evidence over the past 15 years has now firmly established sensory signaling between epidermal keratinocytes and small caliber axons of primary afferents.^4,13,41^ Here, we present the observation of GluA4 AMPAR in mouse and human keratinocytes. Reverse transcription PCR confirmed Gria4 mRNA expression in FACS-isolated mouse keratinocytes. In addition, in situ hybridization conducted in skin biopsies from human control volunteers confirmed GRIA4 mRNA expression in epidermal keratinocytes. GluA4 expression was also explored in the epidermis of patients with AD or PHN. Intense GluA4-IL expression was present in the parakeratosis plaques in SC of biopsies from afflicted skin areas in patients with AD, and this increase coincided with an intense TSLP expression. By contrast, GluA4-IL and mRNA were diminished in hypersensitive skin areas from PHN patients. In organotypic cultures, capsaicin, and CFA, 2 proinflammatory algogenic agents typically used in experimental pain models triggered a decrease in GluA4-IL and mRNA expression. Thus, GluA4 keratinocyte expression was differentially modulated under naturally occurring specific disease conditions involving epidermal pathology and sensory abnormalities.

Our studies of the mouse epidermis showed keratinocyte GluA4-IL in distinct locations (Fig. 1A–C), suggesting that multiple sources of glutamate may exist for the GluA4 AMPAR. Genever et al.^20^ documented expression of the high-affinity glutamate transporter EAAT2 in differentiating suprabasal keratinocyte layers, whereas a different transporter EAAT3 was expressed in the SB, which contains proliferative keratinocytes. Although keratinocytes themselves are capable of producing and releasing l-glutamate,^17^ primary afferent neurons represent another potential source of glutamate.^12^ Indeed, a recent study by Woo et al.^51^ showed that peripheral nerve endings of primary afferents innervating pelage hairs have vGluT2 and play a role in the organization of the piloneural collar. Woo et al. actively sought cells expressing metabotropic or ionotropic glutamate receptors near vGluT2^+^ nerve endings. Our findings demonstrating GluA4 expression in keratinocytes from the bulge of the hair follicle raise the possibility that these primary afferents modulate keratinocyte function through AMPAR.

GRIA4 mRNA expression has been reported previously in human skin biopsy specimens,^47^ although the identity of the cell type expressing GRIA4 remained obscure. Based on our GluA4-IL in hair follicle bulge stem cells,^5^ we isolated α6(+)/CD34(+)/Sca-1(−) cells and performed RT-PCR and Sanger sequencing, thereby confirming that these cells express Gria4/GluA4. GluA4-IL was prominently observed in the inner layer of the bulge, which includes proliferating descendants of the outer root sheath stem cells. The existence of Ca^2+^ permeable AMPAR subunits in the inner layer of the bulge suggests that AMPAR could participate in Ca^2+^-induced differentiation of keratinocytes.^23^ However, unsorted keratinocyte preparations yielded at least one other RT-PCR product (Fig. 1E). The sequence identity of this additional band was not pursued; however, it is likely that a splice variant of *Gria4* may also exist in other cells besides the α6(+)/CD34(+)/Sca-1(−) keratinocytes. In this regard, neuronal *Gria4* isoforms demonstrate diversity through alternative splicing^46^ and mRNA editing.^7^

Expression of GRIA4/GluA4-IL was also found in human newborn foreskin samples. GluA4-IL in human foreskin sections was weak in the SB, and gradually increased in differentiated layers until reaching the highest intensity in the upper SG, then faded out again in the SC. This expression pattern matches approximately with the Ca^2+^ gradient described in mammalian epidermis,^18^ supporting the view of GluA4 playing a role in keratinocyte differentiation. Comparing the GluA4-IL of mouse and human skin samples, we note some similarities and some differences between these results. For example, GluA4-IL of both mouse and human skins showed membranous localization, was generally lower in less differentiated keratinocytes (SB keratinocytes from glabrous skin, or CD34(+) cells in the case of mouse hairy skin), and correlated with differentiation of keratinocytes (suprabasal keratinocytes in glabrous skin, or cells from the inner layer of the bulge of the hair follicle).^26^ However, in the mouse glabrous skin, the more prominent GluA4-IL was found in the SS, whereas in human GluA4-IL was more prominent in the SG.

Our study contributes to a growing body of evidence showing that keratinocytes express a wide variety of molecules implicated in cell proliferation and differentiation, but importantly also in inflammatory and neurosignaling mechanisms.^16,25,27,36^ When these neurotransmitters, neuromodulators, and cytokines stimulate their ionotropic and nonionotropic receptors, keratinocytes release mediators that likely activate receptors on epidermal and juxta-epidermal endings of sensory neurons implicated in nonnoxious mechanical and thermal sensation, as well as noxious itch and nociception. These neurosignaling ligands or cognate receptors expressed by keratinocytes include those typically implicated in neuronal excitation or inhibition. The excitatory receptors include TRP receptors, various G protein–coupled receptors (GPCRs), purinergic ionotropic receptors, voltage-gated ion channels, and their ligands including ATP, calcitonin gene–related peptide, and glutamate.^27,38^ The inhibitory modulators include other GPCRs such as opioid, cannabinoid, and P2Y receptors, as well as ligands including β-endorphin.^14,28^ Importantly, most of the various neurosignaling molecules detected in the epidermis are expressed differentially among the strata of keratinocytes.^14,28^ This suggests that sensory transduction also involves autocrine or paracrine integration among the keratinocytes, in addition to their action on the innervation. Given the natural turnover and differentiation of keratinocyte beginning from proliferation in SB, to extrusion of the nucleus and displacement into SC, it is evident that the differentially stratified signaling systems must be continually reestablished in every new cohort of maturing keratinocytes.

To date, studies regarding cutaneous mechanisms of itch and pain, especially chronic afflictions that are enigmatic and typically untreatable, have focused on the potential varieties and pathologies among epidermal and juxta-epidermal innervation, resulting in considerable debate about whether itch and pain are subserved by separate or confluent types of innervation.^3,22,43^ Several studies have indicated that chronic pain and itch conditions also manifest striking pathologies among the expression patterns of the epidermal keratinocyte neurosignaling molecules that may contribute to the changes in the density and sensitivity of the IENFs.^40,49^ Because glutamate is the main transmitter in small fibers, and is known to be important in keratinocyte signaling, we investigated GluA4 expression among the epidermal keratinocytes in 2 human afflictions with core symptoms of intractable chronic itch (AD) and pain (PHN). In addition to assessing GluA4-IL, we also examined IL for Loricrin as an indicator of keratinocyte maturation,^9^ and TSLP, a keratinocyte produced cytokine which has been extensively implicated in itch.^49,50^ Keratinocytes release TSLP in a calcium-dependent manner, activating a subset of TRPA1(+) sensory neurons to trigger itch responses.^50^ Because TSLP release involves Ca^2+^ flux, and because AMPARs are ionotropic receptors that regulate Ca^2+^ permeability, we sought to assess the expression of GluA4-IL under these pathological conditions.

Our results confirmed previous reports of substantial increases in TSLP-IL expression in AD skin as compared with that in nondiseased skin,^50^ especially as intense expression in the plaques of parakeratosis in SC.^45^ This aberrant TSLP distribution was paralleled by increased and expression of Loricrin-IL in parakeratoses, indicative of accelerated keratinocyte differentiation. GluA4-IL was also consistently and intensely expressed in the plaques of parakeratosis where an increase in intracellular [Ca^2+^] might contribute to pathological keratinocyte differentiation.

By contrast, in 4 of the 5 PHN patients, GluA4-IL was significantly less intense among the epidermal keratinocytes in the biopsies of PHN skin, as compared with GluA4-IL of biopsies of mirror-image contralateral skin, and also when compared with biopsies from normal subjects. Moreover, PHN was not associated with any obvious change in TSLP or Loricrin expression. This decrease in GluA4 may result in Ca^2+^ dysregulation, which could contribute to aberrant neurosignaling patterns observed in PHN skin.^23^

Interestingly, we also observed reductions in GluA4 expression in organotypic stratified keratinocyte cultures, in response to either CFA or capsaicin. Although both agents are able to produce pain and hyperalgesia in intact skin, the consequences of their direct activities on keratinocytes remain unclear. The presence of GluA4 in epidermal keratinocytes is in addition to several other neurosignaling molecules implicated in inhibitory (analgesic) and excitatory (algesic) keratinocyte mechanisms involved in autocrine or paracrine interactions and regulating sensory fiber activity, and which contribute to itch and pain pathways.^4,14,21,25,28,32,42,44,52^ Recently, the direct activation of keratinocyte TRPV1 was shown to evoke aversive behavioral responses;^41^ however, pain and itch could not be explicitly distinguished in that study. Functionally, the alteration of GluA4 subunits among keratinocytes will alter Ca^2+^ sensitivity and signaling dynamics between cutaneous small fibers and IENF that are pruritic or nociceptive. These potential GluA4-mediated signaling differences may contribute to the normal distinctions between itch and pain, as well as primary afferent hyperresponsivity under pathologic conditions.

In summary, this study provides evidence that GluA4-containing AMPARs are expressed in epidermal keratinocytes that human dermatopathies show alterations in the keratinocyte expression levels of GluA4-containing AMPAR, and that algogenic substances can directly regulate the production of GluA4 in keratinocytes. Our findings provide an impetus for future functional studies of AMPAR channel activity and its biological consequences in keratinocytes.

## Conflict of interest statement

The authors have no conflicts of interest to declare.

This work was supported by National Institutes of Health Grants DA027460, DA036826 to J.A.M. and R21AG044633 to D.M.O., and by the Pilot and Feasibility program from the Skin Disease Research Center at Columbia University (P30AR044535) and P30 CA008748 (CCSG) at Memorial Sloan Kettering Cancer Center. Organotypic keratinocyte research was funded, in part, by a New York State Capital Alliance Program (NYCAP) grant awarded to P.J.A.

D.C., T.I., and M.C. contributed equally.

## Supplementary Material

SUPPLEMENTARY MATERIAL

## Appendix A Supplemental Digital Content

Supplemental Digital Content associated with this article can be found online at http://links.lww.com/PR9/A1.
