# Supplementary material for: Identification of an epidermal keratinocyte AMPA glutamate receptor involved in dermatopathies associated with sensory abnormalities
Source: Pain Rep. 2016 Oct 31;1(3):e573. doi: 10.1097/PR9.0000000000000573 (PMC5305184; doi:10.1097/PR9.0000000000000573)
Supplement: SUPPLEMENTARY MATERIAL [file painreports-1-e573-s001.docx]

Supplementary Information for

**Identification of an epidermal keratinocyte AMPA glutamate receptor
regulated in dermatopathies associated with sensory abnormalities**

David Cabañero^1,2^♯, Takeshi Irie^3^♯, Marta Celorrio^1^♯, Christopher Trousdale^1^,
David M. Owens^4,5^, David Virley^6^, Phillip J. Albrecht^7^, Michael J. Caterina^8^,
Frank L. Rice^7^, Jose A. Morón^1^*

* Corresponding Author: Dr Jose A. Morón, Department of Anesthesiology, Washington University School of Medicine, Washington University Pain Center

E-mail: [jmoron-concepcion@wustl.edu](mailto:jmoron-concepcion@wustl.edu); Tel: 314-362-0078

Supplementary Figure 1. Negative controls for the specificity of GluA4 antibody staining in mouse and human skin specimens.

Supplementary Figure 2. Sequencing of mouse CD34+ keratinocyte *Gria4* RTPCR.

Supplementary Figure 3. Comparisons of GluA4-IL in skin biopsies from 5 normal subjects and 5 age-matched postherpetic neuralgia (PHN) subjects.

**Supplementary Figure 1**

**Supplementary Figure 1. Negative controls for the specificity of the GluA4 antibody staining in mouse and human skin specimens**.

**S1a**. Representative confocal images in the absence (control) or presence (GluA4) of the c-terminus GluA4 antibody (anti-GluA4C) in specimens of mouse glabrous and hairy skin.

**S1b**. Representative confocal images in the absence (control) or presence (GluA4) of the c-terminus GluA4 antibody (anti-GluA4C) in specimens of human forearm and back skin.

Scale bar = 20μm

**Supplementary Figure 2.** **Mouse CD34+ keratinocyte *Gria4* RTPCR sequencing.**

**S2a**. The TOPO cloned *Gria4* ex18-ex19 RTPCR product insert sequence was BLAT aligned against the mouse genome, using the UCSC genome browser. Note that the *Gria4* locus is on the antisense strand, with exon 18 represented on the right, and exon 19 on the left.

**S2b**. The sequence of the RTPCR product at the splice junction between exon 18 and exon 19 is identical to other RefSeq entries of *Gria4* splice variants employing these exons. The sequence in black above the chromatogram is the sequence of the aligned region from the UCSC genome.

**Supplementary Figure 3**

**Supplementary Figure 3.** Comparisons of GluA4-IL in skin biopsies from 5 normal subjects and 5 age-matched postherpetic neuralgia (PHN) subjects. All biopsies are labeled against GluA4N, except PHN4 which is against GluA4C. The intensity of GluA4-IL is comparable in mirror image right and left side biopsies from each of the normal subjects. The intensity of GluA4-IL is consistently lower in the PHN biopsies than in the mirror image unafflicted biopsies.
